# Immunization with Virus-Like Particle Vaccine Protects Rabbits against Hepatitis E-3 Virus Infection

**DOI:** 10.3390/v14071432

**Published:** 2022-06-29

**Authors:** Hyeon-Jeong Go, Byung-Joo Park, Hee-Seop Ahn, Sang-Hoon Han, Dong-Hwi Kim, Eu-Lim Lyoo, Da-Yoon Kim, Jae-Hyeong Kim, Joong-Bok Lee, Seung-Yong Park, Chang-Seon Song, Sang-Won Lee, Yang-Kyu Choi, In-Soo Choi

**Affiliations:** 1 Department of Infectious Disease, College of Veterinary Medicine, Konkuk University, 120 Neundong-ro, Gwangjin-gu, Seoul 05029, Korea; goluffy@konkuk.ac.kr (H.-J.G.); twilight@konkuk.ac.kr (B.-J.P.); frequency0@konkuk.ac.kr (H.-S.A.); silverxi@konkuk.ac.kr (S.-H.H.); opeean0@konkuk.ac.kr (D.-H.K.); eulimx0x1230@konkuk.ac.kr (E.-L.L.); kimda68@konkuk.ac.kr (D.-Y.K.); mirine2u@konkuk.ac.kr (J.-H.K.); virus@konkuk.ac.kr (J.-B.L.); paseyo@konkuk.ac.kr (S.-Y.P.); songcs@konkuk.ac.kr (C.-S.S.); odssey@konkuk.ac.kr (S.-W.L.); 2 KU Center for Animal Blood Medical Science, Konkuk University, 120 Neungdong-ro, Gwangjin-gu, Seoul 05029, Korea; 3 Konkuk University Zoonotic Diseases Research Center, Konkuk University, 120 Neungdong-ro, Gwangjin-gu, Seoul 05029, Korea; 4 Department of Laboratory Animal Medicine, College of Veterinary Medicine, Konkuk University, 120 Neundong-ro, Gwangjin-gu, Seoul 05029, Korea; yangkyuc@konkuk.ac.kr

**Keywords:** hepatitis E virus, virus-like particle, rabbit, antibody, liver fibrosis, cytokine

## Abstract

Here, rabbits were immunized with a virus-like particle (VLP) vaccine prepared by expressing 239 amino acids of the swine hepatitis E virus (HEV)-3 capsid protein using a baculovirus system. Thirty specific-pathogen-free rabbits were divided into five groups (negative and positive control and 10, 50, and 100 μg VLP-vaccinated). Positive control group rabbits showed viremia and fecal viral shedding, whereas rabbits vaccinated with 10 μg VLP showed transient fecal viral shedding, and rabbits vaccinated with 50 and 100 μg VLP did not show viremia or fecal viral shedding. Serum anti-HEV antibody titers increased in a dose-dependent manner. Anti-HEV antibody titers were significantly higher (*p* < 0.05) in 100 μg VLP-vaccinated rabbits than in the negative control rabbits at week 4. Anti-HEV antibody titers were significantly higher in 50 and 10 μg VLP-vaccinated rabbits than in the negative control rabbits at weeks 8 and 11, respectively. Serum IFN-γ and IL-12 levels were significantly higher (*p* < 0.01) in rabbits vaccinated with 50 and 100 μg VLP than in the negative control rabbits at weeks 4 and 6. Liver tissues of 50 and 100 μg VLP-vaccinated rabbits displayed significantly less (*p* < 0.05) fibrosis than those of the positive control rabbits. The prepared VLP vaccine demonstrated dose-dependent immunogenicity sufficient for inducing anti-HEV antibody production, thus protecting rabbits against swine HEV-3.

## 1. Introduction

Hepatitis E virus (HEV) belongs to the families *Hepeviridae*, *Orthohepevirus*, and *Orthohepevirus A* and is a non-enveloped virus with a 7.2-kb positive single-stranded RNA [1]. The virion has a spherical and icosahedral form with a diameter of approximately 27–34 nm [2]. HEV can be divided into eight genotypes according to genetic analysis of viruses obtained from human and animal samples [3]. To date, among the eight genotypes, HEV-1, -2, -3, -4, and -7 infections have been reported in humans. Detection of HEV-1 and -2 has mainly been reported in countries where water supply and sewage facilities are suboptimal [4]. The zoonotic transmission of HEV-3 and -4 has been reported in developed countries from eating undercooked animal products, such as raw pork liver sausage and wild boar meat [5,6].

The HEV genome consists of three open reading frames (ORFs) [7]. ORF1, located at the 5′ end of the genome, encodes nonstructural proteins [8]. ORF2 encodes a viral capsid protein that protects the viral genome and interacts with the host cells [9]. Many studies have suggested that the protein encoded by ORF3 is required for the release of viral particles and the production of quasi-enveloped particles [10,11]. Owing to the difficulty in establishing HEV cell culture systems, research regarding HEV vaccine development has focused on virus-like particle (VLP) vaccines using capsid proteins. VLP vaccines can present antigens by mimicking viral capsid proteins. VLPs are safe vaccine candidates because they do not have a viral genome.

There have been many attempts to evaluate the efficacy of a vaccine by expressing HEV VLPs using the *Escherichia coli* expression system and the baculovirus insect cell expression system [12,13,14,15,16]. Using the *E. coli* expression system, a large amount of protein can be obtained quickly at a low production cost. However, as a prokaryotic expression system, the *E. coli* system cannot translate multiple proteins and post-translational modifications. The baculovirus expression system can produce particles that are morphologically and antigenically similar to the native virus particle through post-translational modification. However, this system takes a relatively long time to produce particles compared to the *E. coli* expression system [17,18]. The VLP vaccine, consisting of 239 amino acids manufactured using the *E. coli* system, has already been verified for its antigenicity, immunogenicity, and protectivity against HEV genotypes 1 and 4 in primates [12]. In addition, the HEV 239 VLP has been confirmed to be effective in humans through several clinical trials and was released as a preventive measure in China in 2012 [13,16,19]. The immunogenicity and protective efficacy of the HEV VLP vaccine produced using the baculovirus insect cell expression system, based on 496 amino acids of ORF2, were established in experiments using primates [14,20]. Clinical trials in humans have indicated that the baculovirus-expressed 56-kDa HEV vaccine was effective in preventing the occurrence of hepatitis E [15].

Research on HEV VLP vaccines has mainly focused on antigenicity and humoral immune responses, such as immunogenicity and vaccine protection efficacy against HEV [12,13,15]. Studies on whether the T cell response is included in the protection induced by VLP against HEV are lacking and would be valuable. In the present study, based on swine HEV-3 ORF2 sequences, HEV-3-239-VLP was produced using a baculovirus insect cell expression system. The produced vaccine could sufficiently defend rabbits against swine HEV and induce a Th1-type T cell immune response. HEV-3-239-VLP is considered a candidate HEV vaccine for future use.

## 2. Materials and Methods

### 2.1. VLP Vaccine and Virus

The VLP vaccine described herein was used in a previous study [21]. Briefly, after expressing HEV-3-239 VLP in the baculovirus insect cell system, the collected VLPs were purified using a discontinuous sucrose gradient in 10–50% sucrose solutions. The protein collected from the sucrose gradient layer was dialyzed with 20 mM ammonium bicarbonate solution (Sigma-Aldrich, St. Louis, MO, USA) to remove the unassembled VLP. The protein concentration of purified VLP was measured using the Pierce™ BCA Protein Assay Kit (Thermo Fisher Scientific, Agawam, MA, USA). VLP vaccine (10, 50, and 100 μg/mL) was inoculated into rabbits in the low-, medium-, and high-dose vaccinated groups, respectively. Aluminum hydroxide (2 mg) was added as an adjuvant to each dose of the vaccine. Genotype 3 HEV detected in pig feces was used for virus challenge inoculation [22]. HEV was quantified using reverse transcription real-time polymerase chain reaction (RT-qPCR) (Table 1).

### 2.2. Animals, Immunization, and Challenge Schedule

Thirty 12-week-old female specific-pathogen-free rabbits were purchased from SAMTAKO (Osan, South Korea). All animal experiments were conducted in accordance with the guidelines of the Institutional Animal Care and Use Committee of Konkuk University, South Korea (KU19071). Prior to conducting the experiment, the presence of HEV and anti-HEV antibodies was verified in the feces and serum samples obtained from all rabbits. Rabbits were divided into five groups, namely the negative control; positive control (only HEV-challenged); and 10 μg, 50 μg, and 100 μg VLP-vaccinated groups, with six rabbits in each group. The experiment was conducted for 12 weeks. Rabbits in the vaccine groups received the 1st and 2nd vaccine dose at week 0 (W0) and week 2 (W2), respectively. The vaccination schedule was determined based on our and other previous studies [21,23,24]. At week 4 (W4), the rabbits in the positive control and three vaccine groups were challenged with genotype 3 swine HEV at a concentration of 10^6^ genome copies/mL in 1% bovine serum albumin (Sigma-Aldrich). The HEV-3-239 VLP vaccine was administered into the biceps femoris, and swine HEV-3 was injected into the ear vein. All rabbits were euthanized with potassium chloride after zolazepam administration at week 12.

### 2.3. HEV RNA PCR and Anti-HEV Total Antibody Analysis

Blood collected from the rabbits was centrifuged to separate the serum (2000× *g*, 20 min). Feces collected from the rabbits were dissolved in phosphate-buffered saline (PBS) at a ratio of 1:10 and released using a vortex mixer. The supernatant containing HEV was separated from the fecal solution using centrifugation (2000× *g*, 20 min). Liver samples from the sacrificed rabbits were cut into small pieces, and the excised liver tissue was prepared as a suspension using a cell strainer and PBS containing RNase. All serum, fecal supernatant, and liver samples were stored at −70 °C. HEV RNA was extracted from the collected serum and fecal supernatant using the Patho Gene-spin DNA/RNA Extraction kit (iNtRON, Seongnam, Korea). Nested RT-PCR was performed with two pairs of primers [25] and Maxime™ PCR PreMix (i-starTaq; iNtRON). The total anti-HEV antibody titer was measured using an HEV total antibody ELISA kit (Beijing Wantai Biological, Beijing, China) according to the manufacturer’s instructions.

### 2.4. Alanine Aminotransferase (ALT) and Aspartate Aminotransferase (AST) Levels Assessment

ALT and AST levels were measured using serum isolated from the blood collected weekly from the rabbits. The values were measured using the UV assay according to the procedures established by the International Federation of Clinical Chemistry and Laboratory Medicine using a chemistry analyzer BS490 (NEODIN Biovet Laboratory, Seoul, Korea). The test was repeated twice, and the average value was calculated. 

### 2.5. Cytokine Level Assessment Using ELISA

The levels of interleukin (IL)-10, IL-12, tumor necrosis factor-α (TNF-α), and interferon-γ (IFN-γ) were measured in serum collected at weeks 0, 2, 4, 6, 8, 10, and 12 using commercially available ELISA kits—rabbit IL-10 ELISA kit (Cusabio, Houston, TX, USA), rabbit IL-12 ELISA kit (Fine Test, Wuhan, Hubei Province, China), rabbit TNF-α DuoSet ELISA (R&D System, Minneapolis, MN, USA), and rabbit IFN-γ Do-It-Yourself ELISA (Kingfisher, MN, USA)—according to the manufacturer’s instructions.

### 2.6. Histopathological Examination

Livers obtained from rabbits euthanized at week 12 were fixed with 10% neutral buffered formalin and embedded in paraffin. Tissues were stained with hematoxylin and eosin and Masson’s trichrome to assess the overall lesion and fibrosis in the liver. All Masson’s trichrome stained livers were observed at 40× magnification, and the ratio of the fibrotic tissue area to the liver tissue area was calculated using MetaMorph^®^ (Molecular Devices, San Jose, CA, USA).

### 2.7. Statistical Analyses

GraphPad Prism software version 8.0.2 (GraphPad Software, San Diego, CA, USA) was used for statistical analyses and generating graphs. All data are expressed as the means ± SDs of six replicates. The data were analyzed using one-way ANOVA followed by Dunnett’s multiple comparison test as the post hoc test. The statistical significance for each test was set at *p* < 0.05.

## 3. Results

### 3.1. HEV RNA Was Not Detected in the Liver, Serum, and Feces of Rabbits Vaccinated with 100 μg of VLP

The detection of HEV-3 RNA was confirmed in fecal and serum samples collected weekly from rabbits for 12 weeks and in liver samples obtained from euthanized rabbits at week 12. As expected, HEV-3 RNA was not detected in any fecal, serum, or liver suspension samples obtained from rabbits in the negative control group during the experimental period (Table 2 and Table 3). By contrast, rabbits in the positive control group displayed viremia from weeks 6 to 8 and week 10 and fecal shedding from weeks 7 to 9 (Table 2). HEV-3 RNA was detected in feces and serum, except for one rabbit in the positive control group. In addition, HEV-3 RNA was detected in the livers of the three positive control rabbits (Table 3 and Table S1). Transient fecal shedding was observed from week 8 to 9 in 10 μg VLP-vaccinated rabbits, and HEV-3 RNA was detected in the liver of one rabbit. No viremia or fecal shedding was observed in 50 μg VLP-vaccinated rabbits, but HEV-3 RNA was detected in the liver of one rabbit (Table 2, Table 3 and Table S1). By contrast, viremia and fecal shedding were not observed in rabbits (100 μg), and HEV-3 RNA was not detected in the livers of six rabbits in this group (Table 2 and Table 3). These results indicate that vaccination with 100 μg of VLP is sufficient to protect rabbits from HEV infection.

### 3.2. Anti-HEV Antibodies Were Produced after Vaccination with HEV-3-239-VLP in a Dose-Dependent Manner

**Figure 1:**
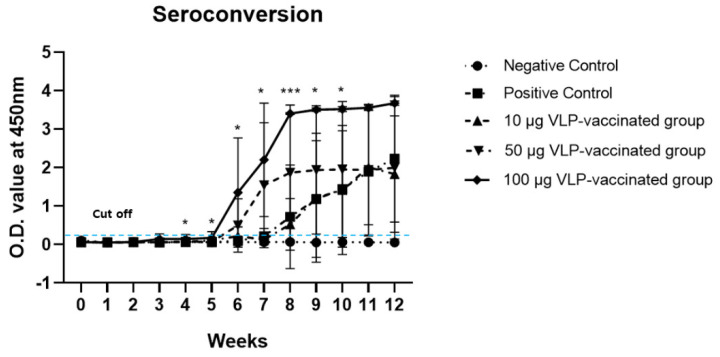
Seroconversion in rabbits immunized with HEV-3-239-VLP. Anti-HEV antibodies of rabbits in 100 µg VLP-vaccinated groups were significantly higher than those of the positive control rabbits from weeks 4 to 10. The antibody titers of rabbits in 50 and 10 µg VLP-vaccinated groups were not significantly higher than those of the positive control. * *p* < 0.05, *** *p* < 0.001.

The presence of anti-HEV antibodies was confirmed in the serum of rabbits from all five groups. As expected, anti-HEV antibodies were not detected in the serum of negative control rabbits. In the serum collected from rabbits in the positive control group, the anti-HEV antibody titer slowly increased, and at only week 12, it was significantly higher (*p* < 0.05) than that of rabbits in the negative control group. The anti-HEV antibody titers of rabbits vaccinated with 100 μg VLP were only significantly higher (*p* < 0.05 or *p* < 0.001) than those of rabbits in the positive control group from weeks 4 to 10 (Figure 1). The antibodies to HEV of rabbits vaccinated with 10 and 50 μg VLP were not significantly higher than those of positive control rabbits.

However, the anti-HEV antibody titers in the serum of 10 μg VLP-vaccinated rabbits increased similarly to the antibody titer of the positive control group and was statistically significant (*p* < 0.05) from week 11 (Figure S1). The anti-HEV antibody titer in the serum of 50 μg VLP-vaccinated rabbits increased significantly (*p* < 0.05) from week 8 (Figure S1). By contrast, in the serum collected from rabbits vaccinated with 100 μg VLP, anti-HEV antibody titers began to increase significantly (*p* < 0.05) from week 4 (Figure S1). These results indicate that anti-HEV antibody production is induced in a dose-dependent manner by HEV-3-239 VLP, which is immunogenic enough to induce antibody production.

### 3.3. ALT and AST Levels of the Positive Control and Three Vaccinated Groups Were Not Significantly Higher than Those of the Negative Control Group

**Figure 2:**
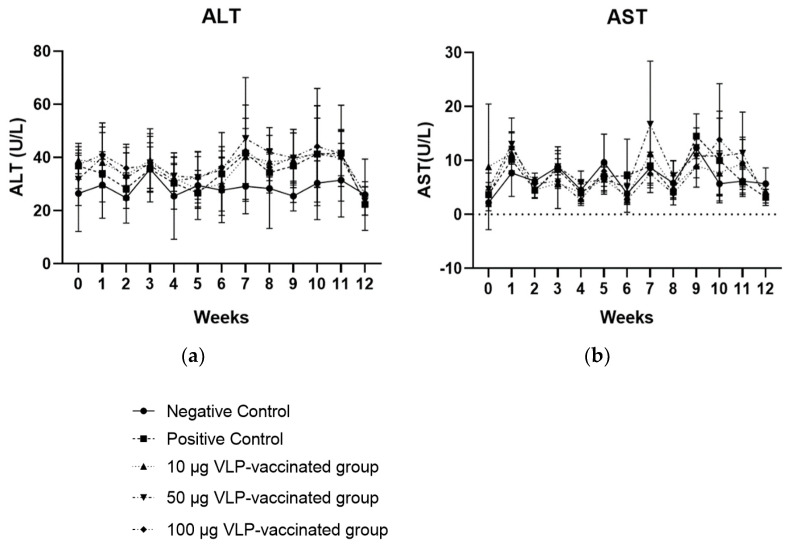
Serum ALT (**a**) and AST (**b**) levels of the rabbits each week. Serum ALT and AST levels of rabbits in the three vaccinated and positive control groups showed no statistical difference compared to those of rabbits in the negative control group.

The ALT and AST levels in the serum of rabbits in the negative control group did not increase. However, the ALT levels of the rabbits in the positive control and three vaccinated groups appeared to increase from week 7, but these were not significantly higher than the ALT levels of the rabbits in the negative control group. ALT levels of all rabbits in the positive control and three vaccinated groups were within the normal range (45–80 U/L; Figure 2a). AST levels in the serum from rabbits in the positive control and three vaccinated groups were maintained throughout the experiment, similar to the AST levels of the negative control group, and there was no significant increase. The AST levels of the rabbits in all groups were within the normal range (35–130 U/L; Figure 2b). In addition, the ALT and AST levels of rabbits that had HEV RNA in their serum, feces, and liver samples were not significantly higher than those of rabbits without viral RNA. Therefore, no significant association was observed between liver enzyme levels and viral detection in rabbits. These results indicated that there was no serious inflammatory response in the livers of positive control and vaccinated rabbits.

### 3.4. Serum IL-12 and IFN-γ Levels Increased after 50 and 100 μg VLP Administration

**Figure 3:**
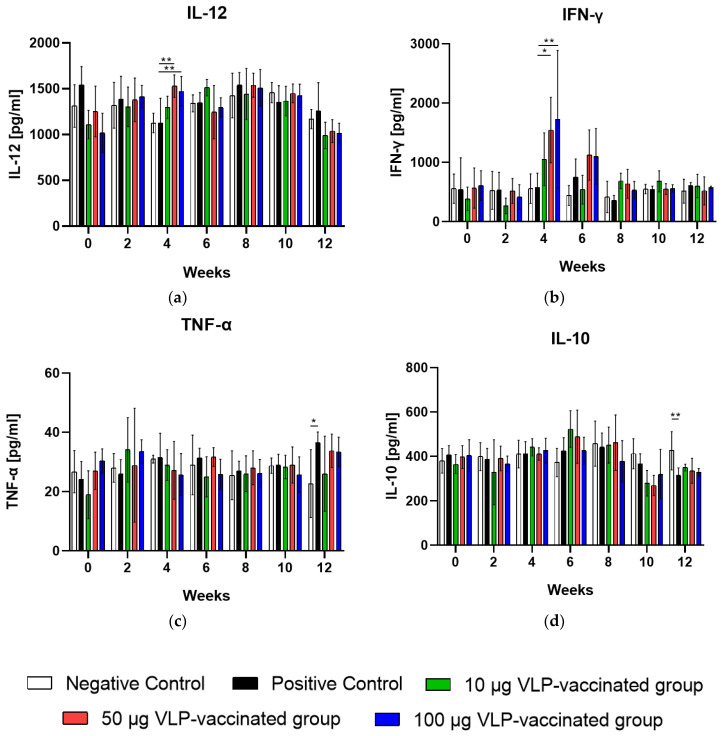
Serum IL-12, IFN-γ, TNF-α, and IL-10 levels were measured in all rabbits every two weeks. (**a**) Serum IL-12 levels of rabbits vaccinated with 50 and 100 μg VLP were significantly higher (*p* < 0.01) than those of the positive control rabbits at week 4. (**b**) Serum IFN-γ levels of rabbits vaccinated with 50 (*p* < 0.05) and 100 μg VLP (*p* < 0.01) were also significantly higher than those of the positive control rabbits at week 4. (**c**) Serum TNF-α levels were not significantly changed between the vaccinated and positive rabbits during the experimental periods. (**d**) Serum IL-10 levels were not significantly changed between the vaccinated and positive rabbits during the experimental periods. Differences were compared between the negative and positive control rabbits and between the positive control and vaccinated rabbits. * *p* < 0.05, ** *p* < 0.01.

The serum IL-12 levels of rabbits vaccinated with 50 and 100 μg VLP were significantly higher (*p* < 0.01) than those of rabbits in positive control groups at week 4. The mean serum IL-12 level of rabbits in the negative and positive control groups was 1126 pg/mL and 1122 pg/mL, respectively, whereas that of the 50 and 100 μg VLP-vaccinated groups was 1529 and 1471 pg/mL, respectively, at week 4 (Figure 3a). Serum IFN-γ levels in rabbits vaccinated with 50 (*p* < 0.05) and 100 μg VLP (*p* < 0.01) increased significantly compared to those in the positive control at week 4. The mean serum IFN-γ levels of negative control rabbits were 557.8 pg/mL at week 4. The mean serum IFN-γ levels of positive control rabbits were 576.8 pg/mL at week 4. The mean serum IFN-γ levels of 50 and 100 μg VLP-vaccinated rabbits increased sharply to 1547 and 1727 pg/mL, respectively, at week 4 (Figure 3b). Serum TNF-α and IL-10 levels of rabbits vaccinated with VLP were not significantly changed to those of positive ones (Figure 3c,d). Collectively, these results indicate that HEV-3-239 VLP vaccination in rabbits induces Th1-type cytokine production.

### 3.5. HEV-3-Induced Liver Fibrosis Was Prevented by 50 and 100 μg VLP

**Figure 4:**
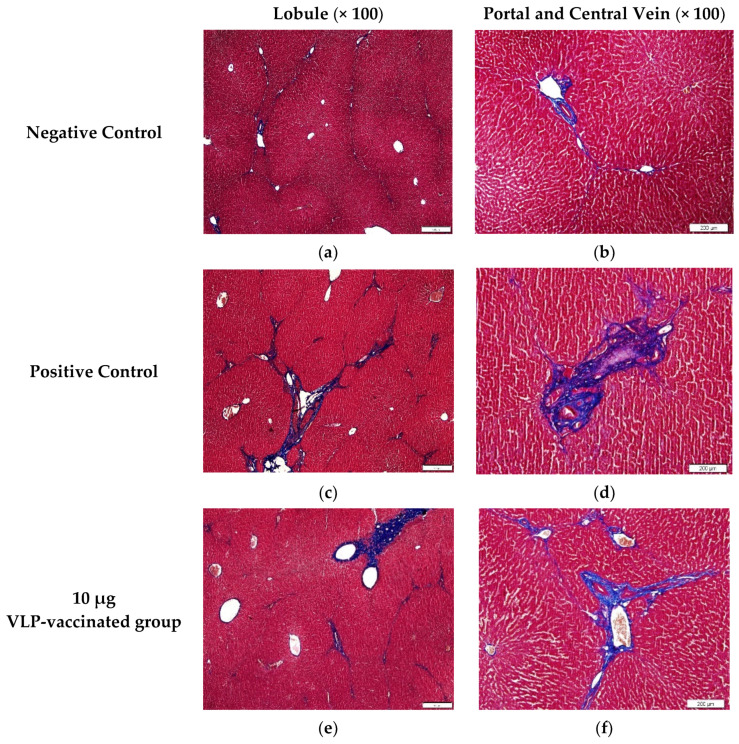

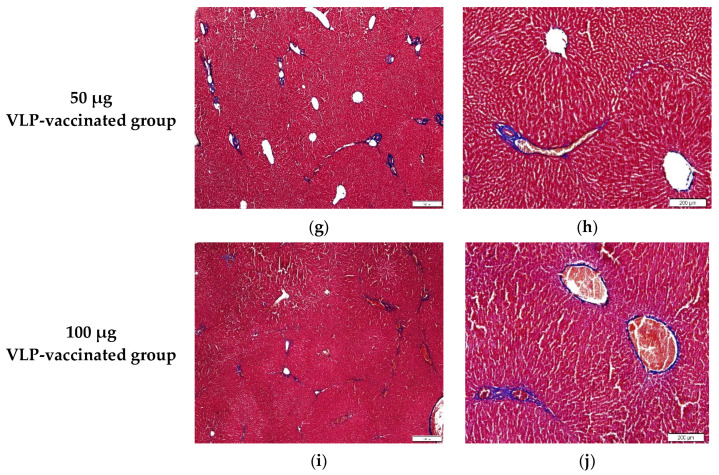
Images of liver tissues stained with Masson’s trichrome. (**a**,**b**) A relatively small amount of fibrotic tissue was found between the hepatic lobules and around the portal and central veins of the negative control rabbits. (**c**,**d**) A large amount of fibrotic tissue was observed between the hepatic lobules and around the portal and central veins of the positive control rabbits. (**e**,**f**) Similar to that in the liver of the positive control rabbits, a relatively large amount of fibrotic tissue was found between the hepatic lobules and around the portal and central veins of 10 μg VLP-vaccinated rabbits. (**g**,**h**) Similar to that in the liver of the negative control rabbits, a relatively small amount of fibrotic tissue was found between the hepatic lobules and around the portal and central veins of 50 μg VLP-vaccinated rabbits. (**i**,**j**) A relatively small amount of fibrotic tissue was found between the hepatic lobules and around the portal and central veins of 100 μg VLP-vaccinated rabbits compared to positive control rabbits.

**Figure 5:**
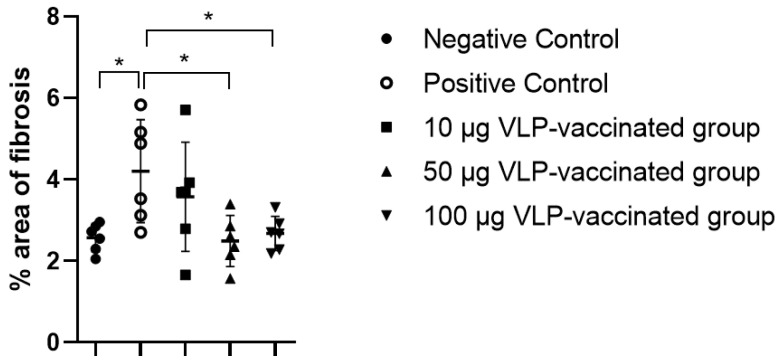
The percentage area of fibrosis in the Masson’s trichrome stained rabbit liver slides. The percentage of fibrotic tissue caused by swine HEV-3 infection in the liver of the positive control rabbits increased significantly compared to that in the liver of the negative control rabbits. The percentage of fibrotic tissue in the liver of rabbits vaccinated with 10 μg VLP appeared to increase but was not statistically significant. Proliferation of fibrotic tissue caused by swine HEV-3 infection was prevented by vaccinating rabbits with 50 and 100 μg VLP. Differences were compared between the negative and positive control rabbits and between the positive control and vaccinated rabbits. * *p* < 0.05.

The livers of the negative control rabbits showed a small number of mononuclear cells. Masson’s trichrome staining was used to determine the degree of fibrosis. Similar to the findings of the livers of the negative control rabbits, a small number of mononuclear cells were observed in the livers of positive control and vaccinated rabbits (Figure S2). Hepatic fibrosis was observed primarily between the hepatic lobules and portal and central veins. As expected, a relatively small amount of connective tissue was observed between the liver lobules and around the portal and central veins in the negative control rabbits (Figure 4a,b). By contrast, a relatively large amount of fibrous tissue was observed between the liver lobules and around the portal and central veins in the livers of positive control rabbits (Figure 4c,d). The livers of rabbits vaccinated with 10 μg VLP had fibrosis similar to those of the positive control rabbits (Figure 4e,f). The livers of 50 and 100 μg VLP-vaccinated rabbits displayed a similar degree of fibrosis to those of the negative control rabbits (Figure 4g–j). The degree of fibrosis was quantified by calculating the area of fibrous tissue relative to the total liver area. The percentage of fibrosis in the livers of positive control rabbits was significantly (*p* < 0.05) higher than that in the livers of negative control rabbits. The percentage of fibrosis in the livers of rabbits vaccinated with 50 and 100 μg VLP was significantly (*p* < 0.05) lower than that in the livers of positive control rabbits. Although the degree of liver fibrosis in rabbits vaccinated with 10 μg VLP was higher than that of negative control rabbits and rabbits vaccinated with the two other concentrations of VLP, the difference was not statistically significant (Figure 5). These results indicated that liver fibrosis in rabbits due to HEV-3 infection could be prevented by HEV-3-239 VLP vaccination.

## 4. Discussion

HEV is known worldwide as the causative agent of acute hepatitis, and the WHO monitors HEV outbreaks [26,27]. HEV infection is usually asymptomatic and self-limiting, but 5–30% of infected patients present with acute hepatitis and non-specific symptoms, such as fever and jaundice. The mortality rate of HEV-infected patients is usually 0.5–4%, but it increases to 20% in pregnant women. In addition, immunocompromised patients are at risk of developing chronic hepatitis following acute hepatitis when infected with HEV [28]. Antiviral drugs, such as ribavirin, are sometimes administered to treat acute HEV infections. Since antiviral drug treatment may fail due to the induction of the mutated HEV virus, preventing infection with vaccinations will be much more effective in controlling HEV [29,30].

P239 (368–606 aa), an extension of the 92 amino acids in the *N*-terminus of the E2 domain of HEV ORF2, can assemble into VLP. The p239 VLP vaccine produced by the *E. coli* system was tested in primates, and its immunogenicity and protective efficacy against HEV infection were verified [12]. In addition, the safety, immunogenicity, and 100% protective efficacy of the HEV 239 VLP vaccine were substantiated in several clinical trials in humans, and it was officially approved in China [13,16,31]. Similarly, the p239 VLP vaccine was developed using a baculovirus system, and the protective efficacy and immunogenicity of the developed VLP vaccine were confirmed by immunizing pigs [21]. Aligning the amino acid sequence of the HEV-3 239 VLP vaccine used in this study with that of other HEV genotypes revealed similar amino acid compositions (Figure S3, Table S2). In this study, immunization of rabbits with the HEV-3-239 VLP vaccine, produced using the baculovirus system, demonstrated the immunogenicity and protective efficacy against swine HEV-3 infection. Furthermore, cytokine production by cellular immune response was also confirmed.

The anti-HEV antibody titer of rabbits vaccinated with the HEV-3-239 VLP vaccine increased in a dose-dependent manner. The titer of anti-HEV antibodies in rabbits vaccinated with 100 μg VLP increased significantly (*p* < 0.05) from week 4 compared to that of the negative and positive control rabbits. Compared to that in positive control rabbits, the titer of anti-HEV antibodies in rabbits vaccinated with 100 μg VLP significantly increased from week 4, suggesting that anti-HEV antibodies were generated following the two vaccine injections. The titer of anti-HEV antibodies in rabbits vaccinated with 50 and 10 μg VLP increased significantly (*p* < 0.05) at weeks 8 and 11, respectively. In this study, we only determined the O.D. values of serum HEV antibodies obtained from rabbits without measuring the end-point titers of antibodies. If the end-point antibody titers were determined, we could identify the exact amounts of antibodies in each serum. It would be a limitation of this study. Fecal shedding was observed for two weeks, from week 8 to 9, in one rabbit vaccinated with 10 μg of the VLP vaccine. However, rabbits vaccinated with 50 and 100 μg VLP did not show viremia or fecal shedding. According to the experimental results, the anti-HEV antibody titers in the serum of rhesus monkeys vaccinated with 10 and 20 μg HEV p239 vaccine, expressed by using the *E. coli* system, increased gradually from week 2, whereas the anti-HEV antibody titers in the serum of monkeys vaccinated with 5 μg VLP increased from week 4. In addition, virus fecal shedding was observed for three weeks and one week in monkeys vaccinated with 5 and 10 μg VLP, respectively [12]. Therefore, the HEV-3-239 VLP used in this study is sufficiently immunogenic to induce the production of anti-HEV antibodies in rabbits, and in line with these results, it prevented HEV infection in a dose-dependent manner. A clinical trial conducted with 30 μg of VLP vaccine made with 239 amino acids of the capsid protein of HEV-1 showed 100% protective efficacy [13]. The vaccine used in the present study almost completely protected rabbits vaccinated with 50 μg of VLP from HEV-3 infection. No serious side effects associated with vaccination were observed in all vaccinated rabbits (data not shown). The VLP could be produced as much as 6 mg from 1 L of SF9 cells infected with the recombinant baculovirus (data not shown). The doses of VLP vaccines for the prevention of human papillomavirus infection are in the ranges of 20–40 μg [32]. We expect a similar dose of VLP would be necessary for the prevention of humans from HEV infection. However, we did not evaluate the vaccine’s efficacy in humans in the present study. This is a limitation of this study. Therefore, further clinical trial studies are necessary to evaluate its optimal dose, efficacy, safety, and costs in humans.

The serum ALT and AST levels of the positive control rabbits infected with swine HEV-3 did not increase significantly and were similar to those of the negative control and vaccinated rabbits. Serum ALT and AST levels of rabbits in all groups were maintained within the normal ranges of 45–80 U/L and 35–130 U/L, respectively, throughout the experimental period. Similarly, ALT levels increased in rabbits infected with swine HEV-3 and rabbit HEV but did not increase significantly and were within the normal range [33,34]. However, elevated ALT levels were seen in the serum of rabbits infected with human and swine HEV-4. This discrepancy may be explained by the difference in the genotype and concentration of the HEV [23,35,36].

In this study, after two VLP vaccinations and one HEV-3 challenge, we confirmed the induction of cytokine production associated with Th1- and Th2-type immune response in the sera of rabbits from all groups. The levels of Th1-type cytokines, IL-12 and IFN-γ, were significantly increased in rabbits immunized with 50 and 100 μg VLP vaccine. By contrast, the serum concentration of IL-10 significantly decreased in all groups at the end of the experimental period. Similarly, IL-12 and IFN-γ levels were significantly increased in the serum of rabbits immunized with the nanogel vaccine based on rabbit HEV [33]. Peripheral blood mononuclear cells were obtained from patients who had recovered after HEV infection and were stimulated with truncated HEV ORF2 protein. These cells displayed significantly increased IFN-γ and IL-12 and decreased IL-10 and IL-4 levels [37]. Th1-type immune response is important for the clearance of hepatitis virus and other RNA viruses [38,39,40,41]. These results suggest that HEV ORF2 protein antigen induces an increase in Th1-type cytokine levels and is related to HEV infection suppression and virus removal. Therefore, it is presumed that not only the humoral immune response but also the cellular immune response is related to the inhibition of HEV-3 infection after HEV VLP vaccination.

In this study, fibrosis of the liver tissue of rabbits infected with swine HEV-3 was determined using Masson’s trichrome staining. The area of fibrotic tissue was calculated as a percentage based on previous studies [42,43,44]. A greater amount of fibrotic tissue was observed between the liver lobules and near the portal and central veins in the livers of positive control rabbits than in the livers of rabbits from other groups. The amount of fibrous tissue was also higher in the livers of rabbits vaccinated with 10 μg VLP than in the livers of rabbits in the other groups. By contrast, a relatively small amount of fibrotic tissue was observed in the livers of rabbits immunized with 50 and 100 µg VLP, similar to that of rabbits in the negative control group. In previous studies, fibrosis was observed in the liver tissue of rabbits infected with swine and rabbit HEVs [34]. Several studies have reported that immunocompromised patients who received organ transplantation developed chronic hepatitis after HEV infection, which then progressed to cirrhosis and liver fibrosis [45,46,47,48]. Thus, fibrosis of liver tissue may be an indicator of HEV infection.

## 5. Conclusions

The HEV-3-239-VLP vaccine, prepared by expressing 239 amino acids of swine HEV-3 capsid protein using a baculovirus system, prevented HEV-3 infection in a dose-dependent manner. Anti-HEV antibodies and Th1-type cytokines may be associated with HEV-3 protection. Fibrosis of the liver tissues could be another indicator of HEV-3 infection. The HEV-3-239 VLP vaccine may be applicable to humans and other animal species in the future to prevent HEV infection.

## Figures and Tables

**Table 1:** Primer and probe set for quantification of the HEV genome.

	Primer Sequence (5′ to 3′)	Position in HEV-3 * (nt)
Forward primer	GGKTRGAATGAATAACATGTY	5122–5142
Reverse primer	GCATAGGCARAARCACGA	5198–5215
Probe	CATCGCCCATGGGATCRCCATG	5148–5169

* Reference strain: swine HEV isolate swKOR-2, complete genome (GenBank Number: FJ426404.1).

**Table 2:** Number of rabbits with HEV RNA detected in feces and sera in each week.

Number of Rabbits															
Group	Weeks Post Inoculation														
	Sample	0	1	2	3	4	5	6	7	8	9	10	11	12	%
Negative control	Serum	0/6	0/6	0/6	0/6	0/6	0/6	0/6	0/6	0/6	0/6	0/6	0/6	0/6	0
	Feces	0/6	0/6	0/6	0/6	0/6	0/6	0/6	0/6	0/6	0/6	0/6	0/6	0/6	0
Positive control	Serum	0/6	0/6	0/6	0/6	0/6	0/6	1/6	2/6	3/6	0/6	1/6	0/6	0/6	50
	Feces	0/6	0/6	0/6	0/6	0/6	0/6	0/6	1/6	3/6	3/6	0/6	0/6	0/6	82.3
10 μg VLP- vaccinated group	Serum	0/6	0/6	0/6	0/6	0/6	0/6	0/6	0/6	0/6	0/6	0/6	0/6	0/6	0
	Feces	0/6	0/6	0/6	0/6	0/6	0/6	0/6	0/6	1/6	1/6	0/6	0/6	0/6	16.7
50 μg VLP- vaccinated group	Serum	0/6	0/6	0/6	0/6	0/6	0/6	0/6	0/6	0/6	0/6	0/6	0/6	0/6	0
	Feces	0/6	0/6	0/6	0/6	0/6	0/6	0/6	0/6	0/6	0/6	0/6	0/6	0/6	0
100 μg VLP- vaccinated group	Serum	0/6	0/6	0/6	0/6	0/6	0/6	0/6	0/6	0/6	0/6	0/6	0/6	0/6	0
	Feces	0/6	0/6	0/6	0/6	0/6	0/6	0/6	0/6	0/6	0/6	0/6	0/6	0/6	0

**Table 3:** Number of rabbits with HEV RNA in livers.

Group	Number of Rabbits with HEV RNA in the Livers
Negative control	0/6 (0%)
Positive control	3/6 (50%)
10 μg VLP-vaccinated group	1/6 (16.7%)
50 μg VLP-vaccinated group	1/6 (16.7%)
100 μg VLP-vaccinated group	0/6 (0%)

## Data Availability

Publicly accessible HEV genotype 1-8 sequences were analyzed in this study. This data can be found NCBI (National Center for Biotechnology Information Advances).

## Supplementary Materials

The following supporting information can be downloaded at: https://www.mdpi.com/article/10.3390/v14071432/s1, Figure S1: Seroconversion in rabbits immunized with HEV-3-239-VLP. Figure S2: Hematoxylin and eosin (H&E) staining of the livers of the rabbits in the negative control, positive control, and three vaccinated groups; Figure S3: Alignment of amino acids sequences corresponding to 239 amino acids of HEV genotype 1 to 8; Table S1: Detection of HEV RNA in the serum, fecal, and liver samples. Table S2: Similarity of 239 amino acid sequences of eight HEV genotypes.
