# Supplementary material for: Immunization with Virus-Like Particle Vaccine Protects Rabbits against Hepatitis E-3 Virus Infection
Source: Viruses. 2022 Jun 29;14(7):1432. doi: 10.3390/v14071432 (PMC9322348; doi:10.3390/v14071432)
Supplement: Supplementary file 1 [file viruses-14-01432-s001.zip › viruses-1765969-supplementary.pdf]

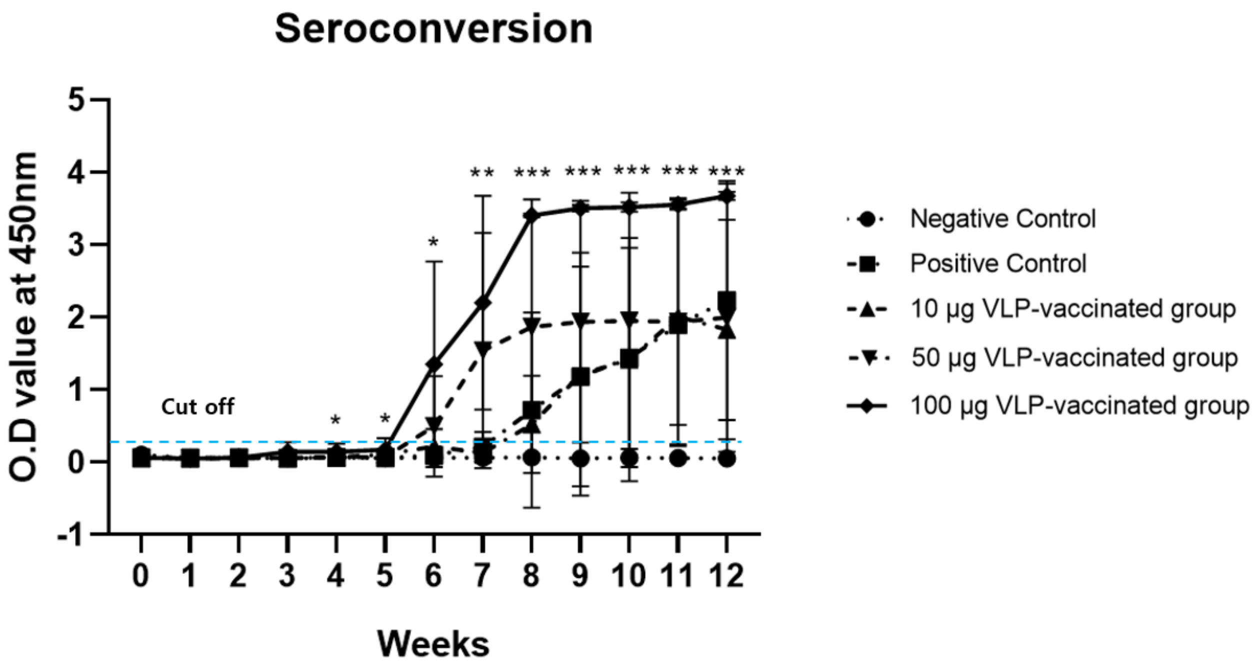

Figure S1. Seroconversion in rabbits immunized with HEV-3-239-VLP. The anti-HEV antibody titer of positive control rabbits increased relatively slowly, and only became significantly higher than that of rabbits in the negative control group at week 12. Anti-HEV antibodies of rabbits in the 10, 50, and 100 μg VLP-vaccinated groups were significantly higher than those of the negative control rabbits from weeks 11, 8, and 4, respectively. * $p < 0.05$, ** $p < 0.01$, *** $p < 0.001$

Table S1. Detection of HEV RNA in serum, fecal and liver samples.

Group	wpi	0	1	2	3	4	5	6	7	8	9	10	11	12	Liver
	No.	F/S	F/S	F/S	F/S	F/S	F/S	F/S	F/S	F/S	F/S	F/S	F/S	F/S	
Negative Control	1	-/-	-/-	-/-	-/-	-/-	-/-	-/-	-/-	-/-	-/-	-/-	-/-	-/-	-
	2	-/-	-/-	-/-	-/-	-/-	-/-	-/-	-/-	-/-	-/-	-/-	-/-	-/-	-
	3	-/-	-/-	-/-	-/-	-/-	-/-	-/-	-/-	-/-	-/-	-/-	-/-	-/-	-
	4	-/-	-/-	-/-	-/-	-/-	-/-	-/-	-/-	-/-	-/-	-/-	-/-	-/-	-
	5	-/-	-/-	-/-	-/-	-/-	-/-	-/-	-/-	-/-	-/-	-/-	-/-	-/-	-
	6	-/-	-/-	-/-	-/-	-/-	-/-	-/-	-/-	-/-	-/-	-/-	-/-	-/-	-
Positive Control	1	-/-	-/-	-/-	-/-	-/-	-/-	-/-	-/-	-/-	-/-	-/+	-/-	-/-	-
	2	-/-	-/-	-/-	-/-	-/-	-/-	-/-	-/-	+/-	-/-	-/-	-/-	-/-	-
	3	-/-	-/-	-/-	-/-	-/-	-/-	-/-	-/+	+/+	+/-	-/-	-/-	-/-	+
	4	-/-	-/-	-/-	-/-	-/-	-/-	-/-	-/-	-/-	-/-	-/-	-/-	-/-	-
	5	-/-	-/-	-/-	-/-	-/-	-/-	-/-	-/-	-/+	+/-	-/-	-/-	-/-	+
	6	-/-	-/-	-/-	-/-	-/-	-/-	-/+	+/+	+/+	+/-	-/-	-/-	-/-	+
10 μg VLP vaccinated group	1	-/-	-/-	-/-	-/-	-/-	-/-	-/-	-/-	-/-	-/-	-/-	-/-	-/-	-
	2	-/-	-/-	-/-	-/-	-/-	-/-	-/-	-/-	+/-	+/-	-/-	-/-	-/-	+
	3	-/-	-/-	-/-	-/-	-/-	-/-	-/-	-/-	-/-	-/-	-/-	-/-	-/-	-
	4	-/-	-/-	-/-	-/-	-/-	-/-	-/-	-/-	-/-	-/-	-/-	-/-	-/-	-
	5	-/-	-/-	-/-	-/-	-/-	-/-	-/-	-/-	-/-	-/-	-/-	-/-	-/-	-
	6	-/-	-/-	-/-	-/-	-/-	-/-	-/-	-/-	-/-	-/-	-/-	-/-	-/-	-
50 μg VLP vaccinated group	1	-/-	-/-	-/-	-/-	-/-	-/-	-/-	-/-	-/-	-/-	-/-	-/-	-/-	-
	2	-/-	-/-	-/-	-/-	-/-	-/-	-/-	-/-	-/-	-/-	-/-	-/-	-/-	-
	3	-/-	-/-	-/-	-/-	-/-	-/-	-/-	-/-	-/-	-/-	-/-	-/-	-/-	+
	4	-/-	-/-	-/-	-/-	-/-	-/-	-/-	-/-	-/-	-/-	-/-	-/-	-/-	-
	5	-/-	-/-	-/-	-/-	-/-	-/-	-/-	-/-	-/-	-/-	-/-	-/-	-/-	-
	6	-/-	-/-	-/-	-/-	-/-	-/-	-/-	-/-	-/-	-/-	-/-	-/-	-/-	-
100 μg VLP vaccinated group	1	-/-	-/-	-/-	-/-	-/-	-/-	-/-	-/-	-/-	-/-	-/-	-/-	-/-	-
	2	-/-	-/-	-/-	-/-	-/-	-/-	-/-	-/-	-/-	-/-	-/-	-/-	-/-	-
	3	-/-	-/-	-/-	-/-	-/-	-/-	-/-	-/-	-/-	-/-	-/-	-/-	-/-	-
	4	-/-	-/-	-/-	-/-	-/-	-/-	-/-	-/-	-/-	-/-	-/-	-/-	-/-	-
	5	-/-	-/-	-/-	-/-	-/-	-/-	-/-	-/-	-/-	-/-	-/-	-/-	-/-	-
	6	-/-	-/-	-/-	-/-	-/-	-/-	-/-	-/-	-/-	-/-	-/-	-/-	-/-	-

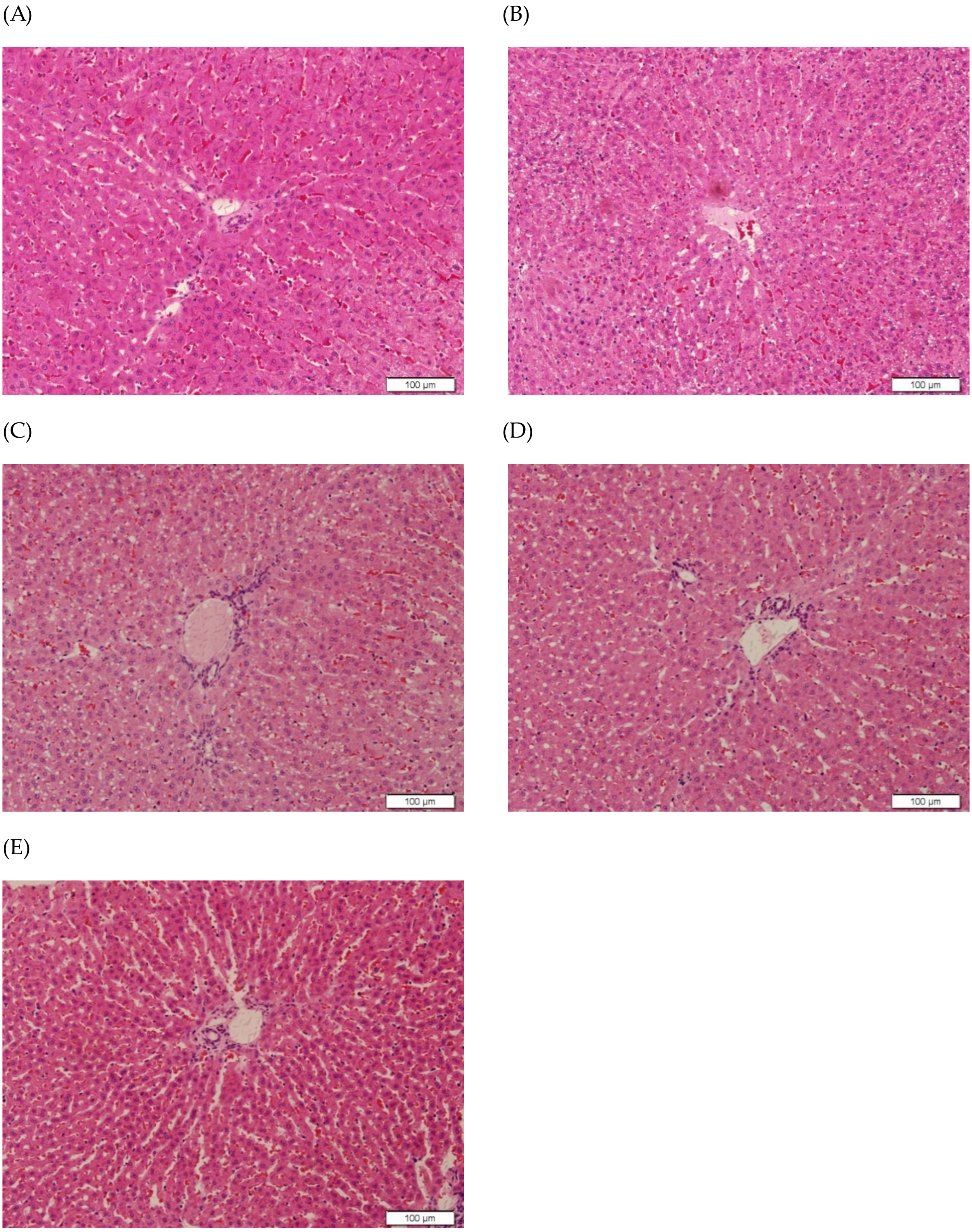

Figure S2. H&E staining of negative control, positive control and three vaccinated group rabbit livers. A small number of mononuclear cells were observed in the livers of rabbits of all groups. (A) Negative control rabbit liver, (B) Positive control rabbit liver, (C) 10 μg VLP vaccinated rabbit liver, (D) 50 μg VLP vaccinated rabbit liver, (E) 100 μg VLP vaccinated rabbit liver.

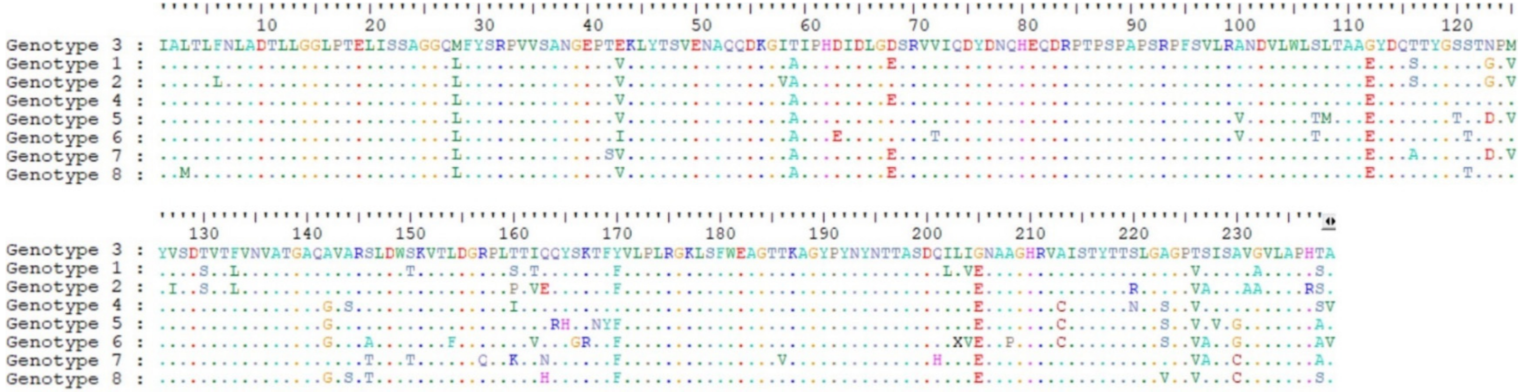

Figure S3. Alignment of amino acids sequences corresponding to 239 amino acids of HEV genotype 1 to 8. The 239 amino acids of the eight genotypes were composed similarly. Genotype 1 (L08816.1), Genotype 2 (M74506.1), Genotype 3 (FJ426404.1, 239 VLP vaccine used this study), Genotype 4 (FJ763142.1), Genotype 5 (AB573435.2) Genotype 6 (AB856243.1), Genotype 7 (KJ496144.1), Genotype 8 (KX387867.1)

Table S2. Similarity of 239 amino acid sequences of eight HEV genotypes.

	Genotype 1 (L08816.1)	Genotype 2 (M74506.1)	Genotype 4 (FJ763142.1)	Genotype 5 (AB573435.2)	Genotype 6 (AB856243.1)	Genotype 7 (KJ496144.1)	Genotype 8 (KX387867.1)
Genotype 3 (FJ426404.1)	91.6%	89.9%	93.7%	90.3%	88.7%	90.7%	92.8%
